# Estimation of Day-Specific Probabilities of Conception during Natural Cycle in Women from Babylon

**DOI:** 10.22074/ijfs.2018.5100

**Published:** 2017-10-14

**Authors:** Hanan Al-taee, Ban J. Edan

**Affiliations:** Department of Physiology, Collage of Medicine, University of Babylon, Babil, Iraq

**Keywords:** Date of Last Menstrual Cycle, Crown-Rump Length, Date of Conception, Ultrasound

## Abstract

**Background:**

Identifying predictors of the probabilities of conception related to the timing and frequency of intercourse in the menstrual cycle is essential for couples attempting pregnancy, users of natural family planning methods, and clinicians diagnosing for possible causes of infertility. The aim of this study is to estimate the days in which the likelihood of conception happened by using first trimester ultrasound fetal biometry in natural cycles and spontaneous pregnancy, and to explore some factors that may affect them.

**Materials and Methods:**

This study is retrospective cohort study, with random sampling. It involved 60 pregnant ladies at first trimester; the date of conception was estimated using: i. Crown-rump length biometry (routine ultrasound examinations were performed at a median of 70 days following Last menstrual period or equivalently 10 weeks), ii. Date of last menstrual cycle. Only women with previous infertility and now conceiving naturally with a certain date of Last menstrual period were selected.

**Results:**

The distribution of conception showed a sharp rise from day 8 onwards, reaching its maximum at day 13 and decreasing to zero by day 30 of Last menstrual period. The older and obese women had conceive earlier than younger women but there was insignificants difference between the two groups (P>0.05). According to the type of infertility, the women with secondary infertility had conceived earlier than those with primary infertility. There was a significant difference between the two groups (P<0.05).

**Conclusion:**

Day specific of conception may be affected by factors such as age, BMI, and type of infertility. This may be confirmed by larger sample size in metacentric study.

## Introduction

Identifying predictors of the probabilities of conception in relation to the timing and frequency of intercourse in the menstrual cycle is essential for couples attempting pregnancy, users of natural family planning methods, clinicians diagnosing for possible causes of infertility, and reproductive epidemiologists (1, 2).

Wide variation in the timing of ovulation has been found in prospective studies related to hormonal and physiological changes such as basal body temperature or ultrasound (3). In pregnant women, the date of conception may be predicted from early fetal growth using sonographic biometry. This method has been proved to be more reliable than last menstrual period for dating the onset of pregnancy (4) and now is considered as a method of choice for dating conception in routine practice (5). However precise knowledge of the timing of conception, however, has important clinical implications: in this context, as for counseling regarding fertility, hormonal ovulation monitoring methods have been made commercially available to help optimizing the chances of conception (6). Timing of ovulation is also important for the follow-up of pregnancies regarding growth monitoring, screening for birth defects and management of delivering (7). Such study has not been conducted in our society. So this study aims to estimate the days in which the likelihood of conception happened by using first trimester ultrasound fetal biometry in natural cycles and spontaneous pregnancy, and to explore some factors that may affect them.

## Materials and Methods

This study is a retrospective cohort study, with random sampling. The study conducted through the period from January 2014-August 2015, involving 60 selected pregnant ladies at first trimester at attending privet ante natal care clinic. Among all patients attended to privet ante natal care clinic in Babylon Province (3000 patients), 312 patients were infertile. Two hindered fifty one of those infertile women fit our inclusion criteria, and only 60 women were randomly selected by excel program. These ladies have already infertility problem and now conceived naturally without any ovulation induction or any hormonal treatment for the past 6 months. All pregnant women have been asked about medical and surgical histories, Physical and ultrasound examination were done in this regard. All the ultrasound examinations performed by single investigator (H.A) using ultrasound machine (Medison, Korea). Date of conception was estimated using crown-rump length biometry [the embryo is measured along its longest axis to obtain the crown-rump length (CRL) measurement] (8). Routine ultrasound examinations were performed at a median of 70 days following last menstrual peroid (LMP) (or equivalently 10 weeks).

Inclusion criteria are as the fillowing: i. Female with history of infertility who have conceived spontaneously without ovulation induction medication, ii. Only women with a certain date of LMP, iii. Singleton pregnancy, and iv. No associated diseases like diabetes mellitus or hypertension. Exclusion criteria are as the fillowing: i. Multiple pregnancy, ii. Diabetes mellitus, protein urea, oligohydrominous, iii. Leaking liquor and vaginal bleeding, iv. Congenital abnormality, v. Uncertain date of LMP, and vi. History of amenorrhea, breast feeding or contraceptive usage or any other hormonal treatment.

The pregnant ladies are subgroup according to the age (more or less than 35 years), body mass index [BMI, normal weight (<24.4 kg/m^2^)], overweight (25-29.9 kg/ m^2^ or obese >30 kg/m^2^) (9) and according to the type of infertility (primary or secondary infertility). The predictive model used to predict date of conception depend on fetal age in days and CRL in mm, expected using the following equation (10).

Fetal age (days)=21.564+2.224 ´ CRL-0.342 ´ ln mm, (CRL) is the measurement of the length of human embryo the top of the head (crown) to the bottom of the buttocks (rump). It is typically determined from ultrasound and can be used to estimate gestational age.

Statistical analysis of data was done by using SPSS version 17. We used independent t test to estimate differences between two groups in continuous variable. Also we used one-way ANOVA to evaluate differences of means among multiple groups. Linear regression analysis used to determine the odds ratio as a measure of the association between factors that affect on date of conception. Results are reported as (mean ± SD) unless indicated. P<0.05, was considered statistically significant (11). All participant signed an informed written consent of their wish of participation, the results of our work. Medical approval by the scientific committee of College of Medicine/University of Babylon.

## Results

Most patients aged less than 35 years (78.3%) and about half of them were in normal weight (53.3%) (Table 1).

**Table 1:** General characteristics of the studied women


Demographic characteristics		n(%)	Total

Type of infertility			60 (100%)
	Primary	32 (53.3%)	
	Secondary	28 (46.7%)	
Age (Y)			60 (100%)
27.25 ± 6.87			
	< 35	47 (78.3 %)	
	>35	13 (21. 7%)	
Body mass index (BMI, Kg/m^2^)			60 (100%)
25.36 ± 4.54			
	Normal	32 (53.3%)	
	Overweight	18 (30.0%)	
	Obese	10 (16.7%)	


The lowest gestational age (GA) values were those estimated by the US method, and the difference with the LMP was significant (P<0.001). The mean ± SD date of conception from LMP was (14.08 ± 5.92) (Table 2).

**Table 2:** Gestational age at by the last menstrual period (LMP) and ultrasound (U/S), and date of conception from LMP


Date in days	Mean ± SD	Lower	Upper	P value

LMP	72.07 ± 9.70	60	105	<0.001^*^^*^
U/S	58.13 ± 9.29	47	78	
Date of conception from LMP	14.08 ± 5.92	8	42	


**Fig.1:**
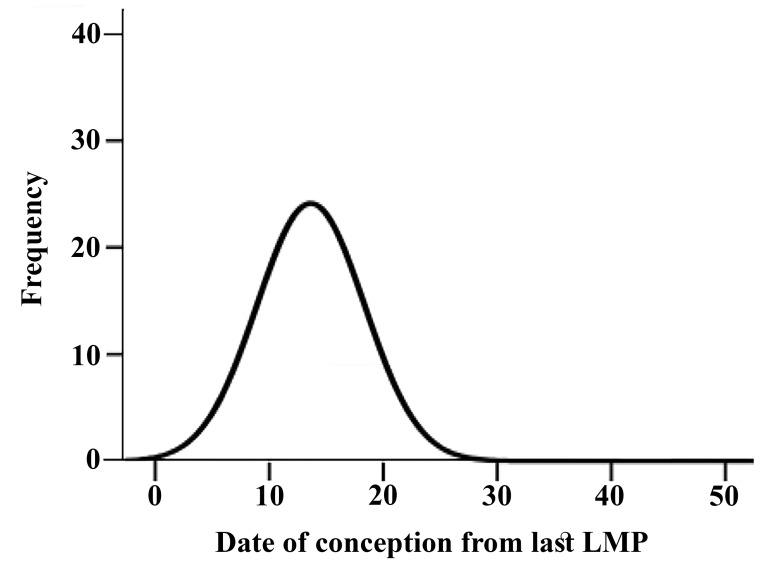
Day-specific frequency of conception in studied women. LMP; Last menstrual period.

This distribution showing a sharp rise from 8 days onwards, reaching its maximum of 18% at 13 days and decreasing to zero by 30 days following LMP (Fig .1).

According to the age, the older women had conceived earlier than younger women but there was insignificant difference between two groups (P>0.05). Women aged >35 year displayed more variation in timing of onset of pregnancy, with an increased likelihood of pregnancies occurring earlier in the cycle (Table 3). Obese women displayed more variation in timing of onset of pregnancy, with an increased likelihood of pregnancies occurring earlier in the cycle (Table 4). According to the type of infertility, the women with secondary infertility had conceived earlier than women with primary infertility. There was a significant difference between two groups (P<0.05).

**Table 3:** Gestational age at by the last menstrual period (LMP) and ultrasound (U/S), and date of conception


Age groups	LMP (mean ± SD)	CRL (mean ± SD)	Date of conception from last LMP(mean ± SD)

<35 Y	73.24 ± 10.25	58.97 ± 9.93	14.50 ± 6.39
>35 Y	67.58 ± 5.45	54.99 ± 5.58	12.50 ± 3.39
P value	0.072	0.189	0.303


CRL; Crown-rump length.

**Table 4:** Gestational age at by the last menstrual period (LMP) and ultrasound (U/S), and date of conception


BMI groups	LMP(mean ± SD)	CRL(mean ± SD)	Date of conception from last LMP(mean ± SD)

Normal	73.35 ± 10.00	58.29 ± 9.26	15.47 ± 7.76
Overweight	71.22 ± 9.88	58.28 ± 10.39	12.84 ± 1.95
Obese	69.33 ± 8.45	57.28 ± 7.91	11.93 ± 1.68
P value	0.505	0.958	0.165


BMI; Body mass index, and CRL; Crown-rump length.

Within the studied group, (46.7%) had secondary infertility. Compared with women with primary infertility, women with secondary infertility displayed more variation in timing of onset of pregnancy, with an increased likelihood of pregnancies occurring earlier in the cycle (Table 5). By linear regression analysis, type of infertility was the most important factor that effects on date of conception, primary infertility associated with delayed date of conception while increase age and BMI associated with prompt date of conception (Table 6).

**Table 5:** Gestational age at by the LMP and ultrasound, and date of conception according types of infertility


Types of infertility	LMP (mean ± SD)	CRL(mean ± SD)	Date of conception from last LMP

Primary	73.73 ± 11.52	58.42 ± 10.59	15.70 ± 7.61
Secondary	70.29 ± 7.04	57.84 ± 7.89	12.40 ± 2.63
P value	0.179	0.816	0.034^*^


LMP; Last menstrual period and CRL; Crown-rump length.

**Table 6:** Linear regression analysis for factors that affect on date of conception


Parameter	B	SE	T	Sig.	95% CI(for B)	
					Lower	Upper

Age (Y)	-0.053	0.153	1.308	0.197	-0.108	0.508
Wt (Kg)	0.03	0.966	0.31	0.975	-1.914	1.974
Ht (m)	0.32	0.870	0.378	0.707	-1.421	2.079
BMI (Kg/m^2^)	-0.37	2.448	-0.150	0.881	-5.292	4.556
Follicle (cm)	0.113	0.402	0.291	0.772	-0.693	0.927
Primary/secondary infertility	1.37	2.046	2.142	0.037	0.266	3.497


Wt; Weight, Ht; Height, BMI; Body mass index, and CI; Confidence interval.

## Discussion

Many research groups had focused to detect the time of ovulation in fertile cycle. Although ovulation generally occurs at around 14 days following the first day of last menses, a wide variation in the timing of ovulation has been found in prospective studies. In pregnant women, the date of conception may be estimated from early fetal growth using sonographic biometry (12). This method has been proved more reliable than last menstrual period for dating the onset of pregnancies (4) and most national guidelines now consider early biometry as the method of choice for dating conception in routine practice (5, 6). Day-specific probabilities of conception are defined as the probability that conception occurs on a given day of the cycle (13), provided that the cycle is fertile. The distribution of date of conception in our study showing a sharp rise from 8 days onwards, reaching its maximum of 18% at 13 days and decreasing to zero by 30 days following LMP which was somehow like the results obtained by Wilcox et al. (14) who found that the maximum probability of conception was reached by day 12.

Women aged >35 displayed more variation in timing of onset of pregnancy, with an increased likelihood of pregnancies occurring earlier in the cycle. These are the same results obtained by Liu et al. (15), Who found that increase maternal age>35 are associated with a shortening of cycle length, and also earlier ovulation.

This study concluded that obese women displayed more variation in timing of onset of pregnancy, with an increased likelihood of pregnancies occurring earlier in the cycle. Minge et al. (16) found that oocytes from the obese mice displayed slower embryo development maintained through the blastocyst stage.

This study demonstrated that women with secondary infertility displayed more variation in timing of onset of pregnancy, with an increased likelihood of pregnancies occurring earlier in the cycle. This result may related to time of oocyte maturation and implantation. Factor that affect oocyte maturation may be related to time of LH surge; the signaling mechanism from the surrounding cumulus cells; and intrinsic oocyte factors. The possibility of an intrinsic oocyte factors remains the most appropriate probability cause for option as the cause of oocyte maturation continuity or arrest (17, 18).

## Conclusion

Day specific of conception may be affected by factors such as age, BMI, and type of infertility. This should be approved by larger in size multicentric study. This study should broaden the perspective of future epidemiologic research in infertility and pregnancy monitoring because of the wider access to retrospective data and the potential bias in prospective studies of ovulation monitoring.

## References

[1] Zhou HB, Weinberg CR (1996). Modeling conception as an aggregated Bernoulli outcome with latent variables via the EM algorithm. Biometrics.

[2] Borawski D, Bluth MH, McPherson RA, Pincus MR (2011). Reproductive function and pregnancy. Henry's clinical diagnosis and management by laboratory methods.

[3] Cole LA, Ladner DG, Byrn FW (2009). The normal variability's of the menstrual cycle. Fertil Steril.

[4] Mustafa G, David RJ (2001). Comparative accuracy of clinical estimate versus menstrual gestational age in computerized birth certificates. Public Health Rep.

[5] American College of Obstetricians and Gynecologists (2009). ACOG Practice Bulletin No.101: Ultrasonography in pregnancy. Obstet Gynecol.

[6] Behre HM, Kuhlage J, Gassner C, Sonntag B, Schem C, Schneider HP (2011). Prediction of ovulation by urinary hormone measurements with the home use ClearPlan Fertility Monitor: comparison with transvaginal ultrasound scans and serum hormone measurements. Hum Reprod.

[7] Stirnemann JJ, Samson A, Bernard JP, Thalabard JC (2013). Day-specific probabilities of conception in fertile cycles resulting in spontaneous pregnancies. Hum Reprod.

[8] Loughna P, Chitty L, Evans T, Chudleigh T (2009). Fetal size and dating: charts recommended for clinical obstetric practice. Ultrasound.

[9] Obesity: preventing and managing the global epidemic: Report of a
WHO Consultation.

[10] Robinson HP (1974). Sonar measurement of fetal crown-rump length as means of assessing maturity in first trimester of pregnancy. Br Med J.

[11] Daniel WW (2010). Probability and t distribution biostatistics: a foundation for analysis in health science.

[12] Mongelli M, Gardosi J (1997). Birth weight, prematurity and accuracy of gestational age. Int J Gynaecol Obstet.

[13] Lynch CD, Jackson LW, Buck Louis GM (2006). Estimation of the dayspecific probabilities of conception: current state of the knowledge and the relevance for epidemiological research. Paediat Perinat Epidemiol.

[14] Wilcox AJ, Dunson D, Baird DD (2000). The timing of the ‘fertile window’ in the menstrual cycle: day specific estimates from a prospective study. BMJ.

[15] Liu Y, Gold EB, Lasley BL, Johnson WO (2004). Factors affecting menstrual cycle characteristics. Am J Epidemiol.

[16] Minge CE, Bennett BD, Norman RJ, Robker RL (2008). Peroxisome proliferatoractivated receptor-gamma agonist rosiglitazone reverses the adverse effects of diet-induced obesity on oocyte quality. Endocrinology.

[17] Howards PP, Cooney MA (2008). Disentangling causal paths between obesity and in vitro fertilization outcomes: an intractable problem?. Fertil Steril.

[18] Chen ZQ, Ming TX, Nielsen HI (2010). Maturation arrest of human oocytes at germinal vesicle stage. J Hum Reprod Sci.

